# *Haemophilus influenzae* Type a Sequence Type 23, Northern Spain

**DOI:** 10.3201/eid2709.204247

**Published:** 2021-09

**Authors:** Maddi López-Olaizola, Amaia Aguirre-Quiñonero, Andrés Canut, José Luis Barrios, Gustavo Cilla, Diego Vicente, José María Marimón

**Affiliations:** Biodonostia Health Research Institute, Infectious Diseases Area, Osakidetza Basque Health Service, Donostialdea Integrated Health Organization, San Sebastián, Spain (M. López-Olaizola, G. Cilla, D. Vicente, J.M. Marimón);; Osakidetza Basque Health Service, Araba Integrated Health Organization, Vitoria-Gasteiz, Spain (A. Aguirre-Quiñonero, A. Canut);; Osakidetza Basque Health Service, Ezkerraldea-Enkarterri-Cruces Integrated Health Organization, Bilbao, Spain (J.L. Barrios)

**Keywords:** *Haemophilus influenzae*, serotype a, ST23, surveillance, respiratory infections, bacteria, Spain

## Abstract

Two consecutive cases of *Haemophilus influenzae* type a sequence type 23 invasive infection in 2 children attending the same daycare in 2019 triggered epidemiologic surveillance of *H. influenzae* infections in northern Spain. Despite the invasiveness potential of this virus strain, we detected no additional cases for 2013–2020.

Since the introduction of the *Haemophilus influenzae* type b (Hib) conjugate vaccine in the infant immunization schedule in 1998, the incidence of invasive *H. influenzae* (Hi) infections in Spain decreased to 0.7 episodes/100,000 population (*1*). Higher incidence rates are observed in children ≤2 years of age (1.88/100,000 population) and adults ≥65 years of age (1.89 cases/100,000 population) (*2*). Invasive disease caused by Hib has nearly disappeared, and most cases are caused by nontypeable strains (*3*). 

Invasive infections caused by *H. influenzae* type a (Hia) are uncommon in Europe, particularly in Spain. However, Hia incidence is as high in other regions as among indigenous communities in North America (*4*) and as has emerged in Brazil during the 2000s (*5*). We describe 2 cases of Hia invasive disease in Gipuzkoa, northern Spain.

Both cases of Hia invasive disease occurred in children in a village with ≈15,000 inhabitants during November 2–3, 2019. The first patient, a 2-year-old boy, was admitted to the pediatric emergency department with good general aspect and persistent low-grade fever without a clear source. The child was not vaccinated according to the routine immunization schedule. Results for pulmonary auscultation and respiratory and cardiac rates were unremarkable, and a chest radiograph showed no abnormalities.

The second patient, a 19-month-old girl, was admitted to the pediatric emergency department with a nonproductive cough and a 39°C fever that was nonresponsive to antipyretics. The infant was vaccinated according to the routine immunization schedule, including Hib vaccination. No dyspnea was observed, and the chest radiograph showed pulmonary infiltrates suggesting pneumonia.

Both children showed increased C-reactive protein, procalcitonin, and white cell counts and had *H. influenzae* grown in the blood culture taken at admission. The boy was treated with ceftriaxone (50 mg/kg/12 h) for 5 days and the girl with ceftriaxone (50 mg/kg/12 h) for 4 days. Both children were discharged without symptoms or sequelae. Neither patient required additional antibiotic treatment after admission. 

Both children attended the same daycare center, where no other children showed symptoms of infection. In Gipuzkoa, no additional cases of Hia invasive infection have been observed since 2013 (Table). However, 1 Hia was isolated 1 week later in the blood-culture of a 51-year-old patient in the adjacent province of Bizkaia.

**Table:** Epidemiologic and microbiological characteristics of *Haemophilus influenzae* invasive isolates, Gipuzkoa, Northern Spain, January 2013–December 2020*

Year	No. isolates†*	Biotype						Capsulated serotypes/ST				Nonencapsulated serotypes (no. isolates)	
		I	II	III	IV	V		a	b	e		NT	ST‡
2013	7	4	2	1					1/ST6			6	41, 368 (2), 388, 996, 2381§
2014	5	1	4						1/ST190			4	105, 249, 1034, 1608
2015	4		3	1								4	40, 155 (2), 2382§
2016	7	1	4	1								6	3, 85, 103, 266, 937, 2383§
2017	5	2	2			1			1/ST6			4	134, 567, 653, 986
2018	9	2		1		4			1¶			6	14, 145, 165, 838, 1472, 2384§
2019	11	1	6	1	2			2/ST23	1/ST995	1/ST760		6	6, 14, 103, 393, 603, 2110
2020	5	2	3									5	143, 183, 280, 334, 349
Total	53	13	24	5	2	5		2	5	1		41	

*NT, nontypeable; ST, sequence type. †Four isolates were not available for microbiological characterization: 1 in 2016, 2 in 2018, 1 in 2019. ‡If the number of isolates of a specific ST was not 1, the number of isolates corresponding to that ST is indicated in brackets. §New ST found in this study. ¶Serotype was determined but the isolate was not available for multilocus sequence typing.

We identified isolates with matrix-assisted laser desorption/ionization time-of-flight mass spectrometry (Beckman Coulter, https://www.beckmancoulter.com). Both case-patients scored >2.000. We biotyped isolates using the API 20E system of bacterial identification and serotyped using multiplex-PCR (*6*) and confirmed serotypes using BD Difco *Haemophilus influenzae* Antisera (Fisher Scientific, https://www.fishersci.com). We performed genotyping by multilocus sequenced typing and pulsed-field gel electrophoresis (PFGE) (Figure) after *Sma*I digestion with the following running conditions: switch angle 120°, 6 V/cm, ramped switch time from 1–30 s over 23 h. The presence of a deletion in the *IS1016-bexA* genes of the capsular operon was studied by PCR as described (*7*). We determined antimicrobial susceptibility by broth microdilution method according to EUCAST version 9.0 guidelines and criteria (EUCAST, https://www.eucast.org).

**Figure:**
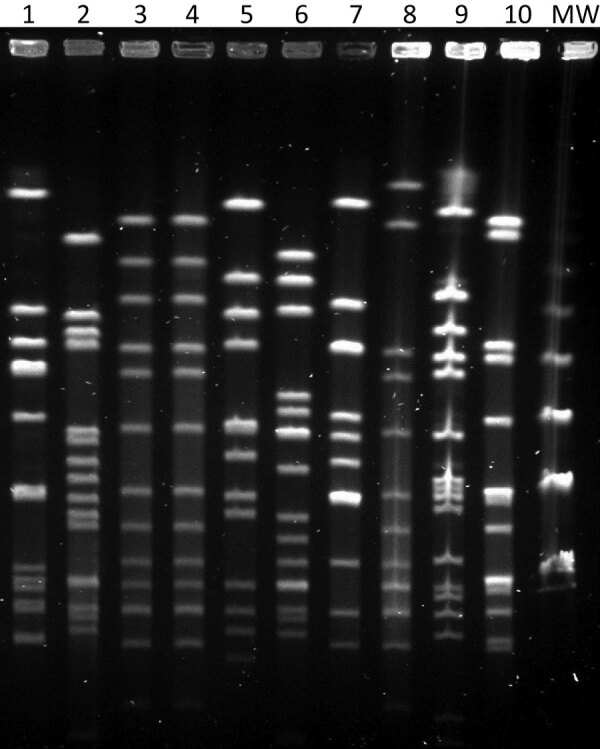
Pulsed-field gel electrophoresis patterns of invasive *Haemophilus influenzae* isolates collected during 2019–2020, Gipuzkoa, northern Spain. Lane 1, ST103; lane 2, ST760; lanes 3–4, ST23 isolates; lane 5, ST393; lane 6, ST6; lane 7, ST995; lane 8, ST2053; lane 9, ST46; lane 10, control isolate ATCC49766; lane MW, 50 kb DNA ladder. ST, sequence type; MW, molecular weight.

The isolates of both children were biotype II, serotype a, sequence type (ST) 23; showed an indistinguishable PFGE pattern; did not show the *IS1016-bexA* partial deletion; and were susceptible to ampicillin, azithromycin, and trimethoprim/sulfamethoxazole. The Hia that was isolated 1 week later in Bizkaia was similar to the 2 previous isolates of Gipuzkoa (biotype II, serotype a, not partial *IS1016-bexA* deletion) but was ST2053 (SLV of ST23) and had a closely related, but not identical, PFGE pattern.

We also characterized all invasive *H. influenzae* isolates reported since 2013 in Gipuzkoa (Table). Of the 48 isolates, 41 (85.4%) were nontypeable and 5 (10.4%) were serotype b; only the 2 cases described in this article were serotype a. All serotype b isolates were biotype I; showed the *IS1016-bexA* partial deletion; and belonged to ST6 (n = 2), ST190, or ST995.

Hia ST23 isolates have been described in different parts of the world, especially in Canada (*4*) and Brazil (*5*). In Europe, Hia ST23 has been found infrequently in Portugal (*8*) and recently in Italy (*9*). The *H. influenzae* multilocus sequence typing database (https://pubmlst.org/organisms/haemophilus-influenzae) lists only 29 ST23 isolates from the United States, Canada, Malaysia, France, and Spain (the 2 isolates in this article), most of them serotype a from invasive diseases.

Hia ST23 isolates from our region and from Canada did not show the virulence-enhancing *IS1016-bexA* partial deletion that has been more commonly associated with increased Hia virulence (*10*). However, isolates from our region only caused a mild and self-limiting infection, as compared with the severe disease observed among native North American Arctic populations that required intensive care unit admission and had notable sequelae (*4*).

As was the case in Italy, transmission of the highly virulent ST23 clone was substantially limited in Gipuzkoa. Although ST23 is a virulent Hia clone, its sustained spread appears to be limited, primarily among indigenous populations of North America. The origin of the isolates described in this article is unknown because Hia ST23 had not been previously described in Spain.
